# Population Structure and Genomic Characterisation of the Ashanti Dwarf Pig of Ghana

**DOI:** 10.3390/ani14050792

**Published:** 2024-03-04

**Authors:** Sethlina Naa Dodua Aryee, Dennis Owusu-Adjei, Richard Osei-Amponsah, Benjamin Matthew Skinner, Esinam Nancy Amuzu-Aweh, Benjamin Ahunu, Anton Enright, Carole Anne Sargent

**Affiliations:** 1 Department of Pathology, University of Cambridge, Cambridge CB2 1TN, UK; aje39@cam.ac.uk (A.E.);; 2 Bristol Medical School, University of Bristol, Bristol BS8 1QU, UK; 3 Department of Animal Science, University of Ghana, Accra P.O. Box LG43, Ghana; doa123@yahoo.com (D.O.-A.); enamuzu-aweh@hotmail.com (E.N.A.-A.); ahunubk@ug.edu.gh (B.A.); 4 School of Life Sciences, University of Essex, Colchester CO4 3SQ, UK; b.skinner@essex.ac.uk

**Keywords:** animal genetic resource, African pigs, genetic diversity, ecological zones, conservation, breeding programmes

## Abstract

**Simple Summary:**

The Ashanti Dwarf pig (ADP), also sometimes referred as the Ashanti Black Forest pig, is the predominant domestic pig breed found in Ghana, West Africa. Although the breed has been traditionally used for subsistence farming and for cultural ceremonies, its potential for commercial farming is gaining attention. There is little information on the genetics of this pig breed which is considered a small breed with an average adult weight of 25–50 kg. In this study, we conducted a population genomic diversity analysis of the Ashanti Dwarf pig and other pig populations within the different regional and ecological zones of Ghana, to ascertain their genetic architecture. The results from this study suggest genetic diversity among the ADP populations found within the different regional and ecological zones of Ghana.

**Abstract:**

There is still limited information on the genomic structure and genetic diversity of African pigs. Genetic diversity studies can contribute significantly to the genetic improvement and conservation of African pigs. This study presents a genetic diversity analysis and population structure of pig breeds in Ghana, with a focus on the Ashanti Dwarf pig (ADP), an indigenous pig breed of Ghana. A total of 167 pigs sampled in Ghana and populations consisting of Ashanti Dwarf pigs (*n* = 106), exotics (mostly European pigs) (*n* = 11), crosses (between indigenous and exotic breeds) (*n* = 44), and unknown breeds (nondescript) (*n* = 6) were genotyped using Porcine SNP60K BeadChip. Moderate heterozygosity levels, ranging from 0.28 for Ashanti Dwarf pigs to 0.31 for exotic pigs (mostly European pigs), were observed. Principal component analysis of the pig populations within Ghana resulted in two distinct clusters of pigs: (i) Northern and (ii) Southern regional clusters. The PCA based on breed also resulted in four clusters: (i) ADPs; (ii) exotics (iii) crossbreeds between ADP and exotics; (iv) unknown breed types. The PCA demonstrated that the clustering was influenced by genetics, geographical location, production systems, and practices. ADMIXTURE-based analysis also showed that the populations within Ghana are admixed. *F*ST analysis revealed SNPs associated with QTLs for traits such as disease resilience and growth among ADP populations within the different regional and ecological zones of Ghana.

## 1. Introduction

The Ashanti Dwarf pig (ADP), just like other local pig breeds in Africa, is small in size, black, and has a long head with a very narrow snout [1,2]. It comes in a variety of colours including black, brown, and black with white or brown patches. This breed is known for its hardiness and adaptation to a wide range of environmental conditions, as well as its resilience to diseases. Molecular characterisation studies have shown that this pig breed has a distinct genetic profile, and it is closely related to other indigenous pig breeds in West Africa [3,4]. The reproductive performance of this breed is considered good with sows having an average litter size of 7–10 piglets [5,6,7,8]. Ashanti Dwarf pigs (ADPs) can breed year-round which makes them suitable for commercial farming. However, good management practices such as providing adequate nutrition, controlling disease outbreak, and providing housing are essential for achieving optimal reproductive performance. The management practices of the Ashanti Dwarf pig are largely based on traditional knowledge, with farmers providing minimal inputs in terms of nutrition and veterinary care. However, there is growing interest in improving the breed’s productivity through better management practices and genetics [3,9]. A study by [10] showed that providing a balanced diet to sows during gestation and lactation increased litter size and piglet weight. Other management practices such as controlling disease outbreaks and improving housing conditions have also been shown to improve productivity [8]. The market potential for the ADP is currently limited due to a lack of awareness about the unique attributes of the breed and the lack of organised markets [11].

Compared to the European pig breeds, the ADP has a compact, muscular body with a black coat that is usually short and bristly. They also have large, erect ears and a long snout with a smaller frame compared to European and Asian pigs. Ashanti Dwarf pigs are also known for their slow growth rate compared to many European breeds. This may be due to the breed’s adaptation to harsh environmental conditions and production practices in Ghana. However, the breed’s slower growth rate can also lead to a leaner meat with less fat content compared to European and Asian breeds [11]. Ashanti Dwarf pigs are typically raised in traditional extensive production systems, where they are allowed to forage and roam freely in open or semi-open areas. Ashanti Dwarf pigs are normally raised at the subsistence level by farmers and mostly used as a source of income in seasons when there is crop failure. Compared to some European breeds, they require less intensive management and may have a lower impact on the environment [2]. However, extensive production systems may also result in lower productivity and may be less suitable for large-scale commercial pig production. Ashanti Dwarf pigs have shown some resistance to diseases such as African swine fever (ASFv), which is a major concern for pig producers in Africa and other regions. Farmers have reported that during outbreaks of ASFv, although the local pigs are affected by the disease, they do not succumb to it, making them very resilient to the ASFv [9].

Despite the documented genetic merits and the huge importance of this pig breed to agriculturalists, its small body size has led to the indiscriminate crossing with imported pig breeds (mostly of European descent) like the Large White and Landrace. Interestingly, although most of the pigs generated through indiscriminate crossbreeding had higher growth rates and increased body size, they had reductions in other genetic merits, such as less ability to handle fibrous diets and reduced resilience to diseases [7]. Aside from the reduction in the genetic merits in this pig breed, there is a high possibility that the ADP is in danger of extinction due to the increasing use of exotic pig breeds and crossbreed strains in the country. The loss of pure-line ADPs could also mean a loss or depletion of favourable alleles in these local pig populations, detrimental to the conservation and sustainable use of this breed. To maintain and improve the potentially rich genetic merits of this pig breed, it is essential that its genomic structure and the specific alleles that contribute to its adaptability or influence its adaptive and productive traits are identified. This study therefore aimed at identifying the population genomic structure and genetic diversity and identifying any strong signatures of selection which harbour important alleles that may be associated with traits of economic importance among Ghanaian pig populations with a focus on the Ashanti Dwarf pig across all the regions of Ghana to promote their effective conservation and sustainable use.

## 2. Materials and Methods

### 2.1. Description of Study Area

**Figure 1:**
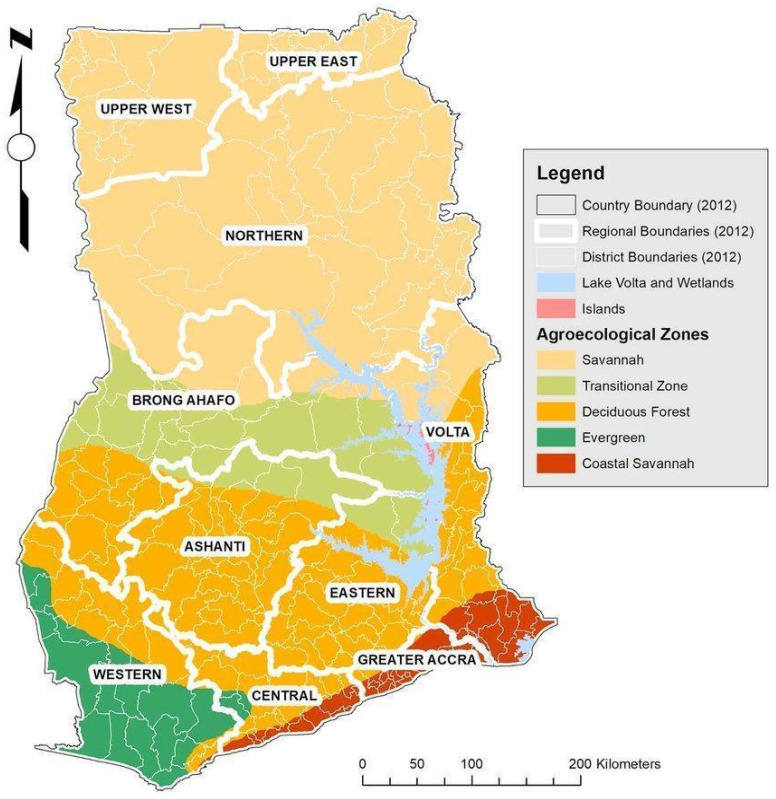
Map of Ghana showing different ecological zones (www.fao.org).

Ghana is a country in Western Africa situated at the shore of the Gulf of Guinea and the Atlantic Ocean. It occupies close to 240,000 km^2^ of land area and is surrounded by Togo in the east, Ivory Coast in the west, Burkina Faso in the north, and the Gulf of Guinea and the Atlantic Ocean in the south. Ghana lies between the latitudes and longitudes of 4°45′ N and 11° N and 1°15′ E and 3°15′ W, respectively. The country at the time of sampling in 2013 was divided into ten administrative regions and the Northern region had the largest land area (70,384 km^2^). The capital city Accra had the least land area (3245 km^2^). There are six ecological zones in Ghana, namely, the Rainforest (evergreen), Deciduous Forest, Guinea Savanna (Transitional), Coastal Savanna, Akwapim Togo ranges, and the Sudan Savanna (Savannah) (Figure 1) [12]. The largest ecological zone is the Guinea Savanna, and it lies in the northern part of the country. The Guinea Savanna zone is characterised by a wooded grassland, which consists of a ground cover of grasses at varying heights [13,14]. It has only one rainfall season (which starts in late April or early May, rises in August to September, and declines in October to November), records an annual rainfall of 1100 mm/year, and has a 27.5 °C annual temperature. The rainy season is usually followed by a long dry period. Farmers in this zone produce annual food plant crops (main crops, i.e., sorghum and maize), cash crops (shea nut and cashew), and livestock (mostly cattle) [15]. The Rainforest records the highest amount of annual rainfall (2200 mm/year). It is characterised by two rainy seasons. The major season lasts for 150 to 160 days and the minor rainy season lasts for a maximum of 100 days. Land in this zone is dominantly used as forest reserves and plantations. Major crops grown in this zone include root crops and plantain. The Deciduous Forest zone is characterised by two main forest types, the moist semi-deciduous forest and dry semi-deciduous forest. The Kwahu plateau is close to the northern boundary of the zone and the southern edge merges into the moist evergreen forest type. Comparatively, the dry season of the forest zone is more defined than the evergreen forest types. The mean annual rainfall ranges from 800 to 2800 mm/year and that of temperature is 26.4 °C in the forest zones [13,16].

### 2.2. Sample Collection and Ethical Approval

A total of 176 ear tissue samples from live local pig populations in Ghana were collected across selected districts in all the then ten administrative regions in 2013 using purposive sampling (animals of the same family were excluded). Out of the 176 ear tissues collected, 80 were originally collected by [3], from the Ashanti region, Greater Accra region, Eastern region, Central region, Upper West region, and Northern regions between August 2013 and October 2013, and the remaining 96 samples were collected from the Upper East region, Brong-Ahafo region, Volta region, and Western region at varying times from October 2015 to February 2016. Permissions were sought from farmers before samples were collected under the guidance and supervision of veterinarians of the Ministry of Food and Agriculture (MoFA), Ghana. The ethical approval for the study was given by the Institutional Animal Care and Use Committee (NIACUC), Noguchi Memorial Institute for Medical Research, University of Ghana, and it was valid until 4 May 2018 [3]. One-hundred and sixty-seven (167) ear tissues samples were genotyped. Although the ADP was the pig breed of major interest in this study, the pig samples included ADP (*n* = 106), locally adapted exotic breeds (*n* = 11), crossbreeds (*n* = 44), and unknown (nondescript) pig breeds (*n* = 6). Each sample collected was put into a test tube which contained RNAlater and transported for DNA extraction at the Biotechnology laboratory of the College of Basic and Applied Sciences, University of Ghana [3].

### 2.3. DNA Extraction, Quality Control, and Genotyping

Total genomic DNA was extracted from ear tissue samples using the Qiagen DNA blood and tissue kit following the manufacturer’s protocol, with minor modifications. Samples were cut into small pieces of about 25 mg (to enable a more efficient lysis), placed in a 1.5 mL microcentrifuge tube, and 180 μL tissue lysis buffer (ATL) was added. An amount of 20 μL proteinase K was added and mixed thoroughly by vortexing. The tissues were then incubated in a water bath at 56 °C and occasionally vortexed over a period of 3 h. An amount of 4 mL of RNase A (100 mg/mL) was added, mixed by vortexing, and incubated for 2 min at room temperature. The solution was then gently mixed again for 15 s before 200 μL Buffer AL was added to the sample and vortexed to mix thoroughly. Subsequently, 200 μL of ethanol (99% concentration) was added and mixed to ensure a homogenous solution. The mixture was then pipetted onto a DNeasy Mini spin column positioned in a 2 mL collection tube provided by the manufacturer and centrifuged at 8000 rpm for 1 min. The collection tubes and the residuals were discarded. The DNeasy Mini spin column was placed in a new 2 mL collection tube provided by the manufacturer, 500 μL Buffer AW1 was added, and the mixture was centrifuged for 1 min at 8000 rpm. The residuals and the collection tubes were again discarded. The procedure was repeated but using 500 μL of Buffer AW2 before centrifuging at 14,000 rpm for 3 min. The final step was the elution, which was conducted by placing the DNeasy Mini spin column in a clean 2 mL microcentrifuge tube and 200 μL of Buffer AE was pipetted directly onto the DNeasy membrane. This was incubated at room temperature for a minute and centrifuged at 8000 rpm for a minute. The flow through contained the extracted genomic DNA which was refrigerated at −80 °C prior to shipping to the laboratory of the Mammalian Molecular Genetics group, Department of Pathology, Cambridge University, for quality control and genotyping. The concentration and quality of total genomic DNA was measured using the Qubit 2.0 fluorometer. The Illumina Porcine SNP 60 K Bead chip with an Infinium HD ultra-assay was used for SNP genotyping following the manufacturer’s protocol, and arrays were scanning using an iScan (Illumina, Cambridge, UK). Data from the images derived from the Illumina iScan were then loaded into Genome Studio for quality control. Using a plug-in which is compatible with GenomeStudio, the genotyped input data were converted into the PLINK data format (Ped file and Map file).

### 2.4. Pig Genome Build

Initial results obtained in this study were run using the pig genome build Sscrofa 10.2 [17] and repeated when Sscrofa11.1 became available [18]. The majority of Sscrofa10.2 SNPs were unambiguously located in Sscrofa11. However, for ‘unmapped’ markers, the entire sequence surrounding each SNP and the probes used on the Illumina build were used to identify unambiguous locations on build 11.1 via BLAST. Of the 61,567 markers on the chip, 59,973 could be assigned a location on the autosomes or sex chromosomes. The remaining markers were found to be either unmatched, on unplaced contigs, or were discovered to have more than one site in the build.

### 2.5. Quality Control

Quality control was conducted in PLINK [19]. All variants with missing genotype call rates above 5% were removed. As this is an exploratory study, variants with minor allele frequency (MAF) ≤ 0.01 in the various populations were also filtered out. This was conducted to reduce bias, since rare alleles may represent pathogenic variants and may also not be helpful for the study size. Tests for individual missingness (--mind 0.05) and the Hardy–Weinberg equilibrium exact test with *p*-value < 0.001 were also conducted in PLINK.

### 2.6. Population Genomic Parameters

#### 2.6.1. Minor Allele Frequency

The minor allele frequencies (MAFs) and the percentage of polymorphic SNPs were estimated after filtering out monomorphic SNPs and variants which had minor allele frequencies (MAFs) ≤ 0.01. The option ‘--freq’ was used to write out minor allele frequency reports.

#### 2.6.2. Heterozygosity and Levels of Inbreeding

The within-population genomic diversity parameters observed heterozygosity (Ho), expected heterozygosity (H_E_), and the inbreeding coefficient (*F*IS) were determined in PLINK using the option --het.

### 2.7. Population Genomic Cluster Analysis

#### 2.7.1. Principal Component Analysis

The principal components of the populations were estimated in PLINK to help evaluate the population stratification with the command ‘--*pca*’. The resulting .eigenvec and .eigenval files were used to generate the PCA plots using the plotly package [20], in R version 3.5 [21].

#### 2.7.2. ADMIXTURE Analysis

To determine the most probable number of ancestry inputs (*K*-value), and to identify what may constitute pure lines of populations, ADMIXTURE [22] was run from K = 1 to K = 10. The input file used to run ADMIXTURE [22] was a ‘*.bed*’ file with an associated ‘*.bim*’ (binary marker information) file and ‘*.fam*’ (pedigree) file generated in PLINK [19].

#### 2.7.3. FST Analysis

*F*ST (fixation index) which measures the level of genetic differentiation between and within populations was estimated in PLINK using the command ‘--fst’. Genetic differentiations between populations were evaluated based on Wright’s genetic differentiation estimates, i.e., low genetic differentiation, *F*ST = 0.0–0.05; moderate genetic differentiation, *F*ST = 0.05–0.15; moderate-to-high genetic differentiation, *F*ST = 0.15–0.25; and high genetic differentiation, *F*ST = above 0.25 [23].

#### 2.7.4. Gene Ontology (GO) Analysis

String v.11 ([24], https://version-11-0.string-db.org, accessed on 20 May 2021), Ensembl v. 100 ([25], https://www.ensembl.org/index.html, accessed on 19 July 2020), and DAVID v. 6.8 ([26], https://david.ncifcrf.gov/tools.jsp, accessed on 15 January 2021) were used for the GO analysis of the significant genes identified through the *F*ST analysis. Humans or mice were used as reference organisms where pig data were not available.

## 3. Results

### 3.1. Summary of Data

One hundred and sixty-seven (167) animals were selected for genotyping (Table 1). All the individuals (167) passed quality control; no phenotypes were present (phenotypic data were not included in analysis). A total of 61,565 variants were loaded for quality control from the (.bim) file, out of which 59,302 variants passed filters and quality control and 2263 variants were removed as a result of missing genotype data or SNPs deemed to have failed the HWE test (59,302 SNPs out of the 61,565 SNPs passed QC). Hence, the total genotyping call rate was 95.15%.

### 3.2. Inter-Population Genomic Diversity

To study the population genomic diversity of the pigs genotyped, genomic diversity parameters were computed across all the pig populations sampled, based on the originally assigned population classification. Table 2 shows the different populations, the number of individuals for each population (*n*), the percentage of polymorphic SNPs recorded (%SNP > 0.05), the minor allele frequencies across the populations (MAFs), the observed heterozygosity (Ho), the expected heterozygosity (H_E_), and the inbreeding coefficient (*F*_IS_).

### 3.3. Minor Allele Frequency

The Ashanti Dwarf pig populations recorded the highest percentage of polymorphic SNPs (88%) and the lowest (77%) were recorded among the unknown populations. The percentages of polymorphic SNPs recorded in the crosses (ADP × exotics) and exotic populations (mostly European pigs) were 87% and 83%, respectively.

The average minor allele frequencies (MAFs) of individuals of the different populations at each individual SNP loci are represented in Table 2, column 4. Across the populations, the average MAF ranged between 0.23 and 0.26. However, the Ashanti Dwarf and crossbred populations recorded the highest MAF (0.26).

### 3.4. Heterozygosity and Levels of Inbreeding

Heterozygosity was recorded in each of the Ghanaian pig populations. The inbreeding values (*F*_IS_) were lowest in the unknown populations (0.02) and highest (0.15) in the Ashanti Dwarf pig populations (Table 2, column 7).

The observed mean heterozygosity (Ho) ranged from 0.28 to 0.31, while the expected mean heterozygosity (H_E_) ranged from 0.31 to 0.34 (Table 2). The highest observed mean heterozygosity was recorded in the exotic populations, whereas the lowest observed mean heterozygosity was recorded in the Ashanti Dwarf pig populations.

### 3.5. Population Genomic Cluster Analysis

#### 3.5.1. Principal Component Analysis

**Figure 2:**
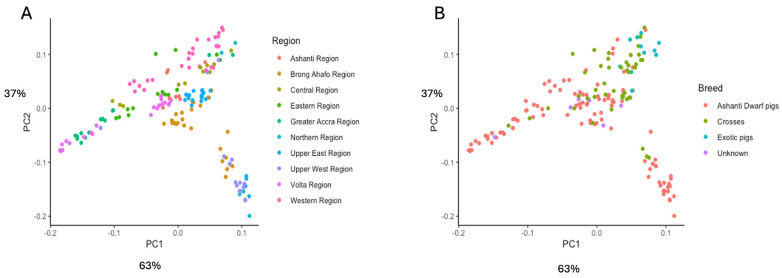
PCA plots showing how the animals studied cluster according to (**A**) regions and (**B**) breed. The predominant association is by region, with PC1 roughly corresponding to the north–south axis of the country.

PC1 and PC2 were plotted against each other first by breed and then by region. PC1 and PC2 explain 63% and 37% of the total variation, respectively (Figure 2). The PCA analysis showed a clear separation of pig breeds in the northern part of the country from the pigs found in the southern part of the country. The ADP populations in the northern part of the country, i.e., Upper West region (UWR), Northern region (NR), Upper East region (UER), and Brong-Ahafo region (BAR), clustered together. ADP populations also found in the south, i.e., Volta region (VR), Greater Accra region (GAR), and Eastern region (ER), also clustered closer to each other. The exotic pig breeds and the crosses were predominant in the Western region (WR) and Central region (CR).

#### 3.5.2. Admixture Analysis

**Figure 3:**
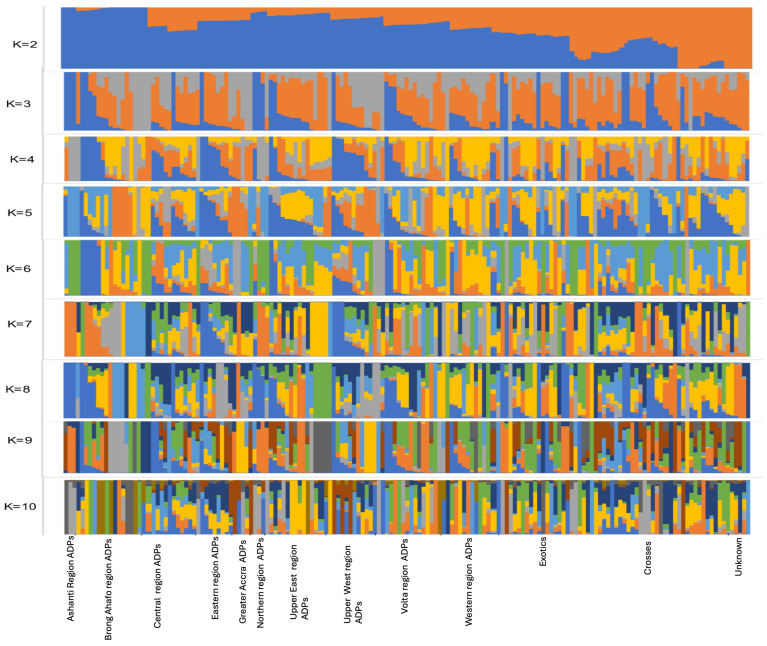
Admixture representation of Ghanaian pig populations. The colour coded blue at K = 3 refers to the ADP populations which are likely to have been admixed with the colours coded orange and grey which are likely to be of European and Chinese descent.

Admixture analysis suggested that the Ghanaian pig populations are indeed of mixed ancestry (Figure 3). At K = 2, the local pig populations clustered showing similar profiles within each subpopulation (all ADP populations, exotics, and crosses) but separate from the unknown populations, indicating that the ADPs found in the different regions may be subpopulations of one ancestral local pig breed influenced by geographical location. The lowest cross-validation error suggested that the most probable number of ancestry inputs was K = 6 (suggesting a stratification of regional ADPs). At K = 3 to K = 10, most of the local pig populations demonstrated increasing levels of admixture (Figure 3). The Ashanti Dwarf pig populations in the Ashanti region, Upper East region, Northern region, Brong-Ahafo region, and Upper West region had individuals that were more similar to each other, remained clustered together from K = 2 to K = 10, and were comparatively less admixed. However, ADPs in the Western and Central regions demonstrated high levels of admixture. This agrees with PCA results which suggest that most of the exotics and crosses are recorded in the Western and Central regions and could account for the increasing levels of admixture observed in the ADP populations sampled from within the Central and Western regions.

#### 3.5.3. Genetic Differentiation, Outlier Loci, and Some Candidate Genes under Selection

Results from the PCA and the admixture analysis suggest regional stratifications within the Ghanaian pig populations. To further understand these differentiations, *F*ST analysis was conducted to discover how the regional and ecological differences may have impacted selection in these pig breeds. The *F*ST values were derived to determine (i) the genetic differentiation within all the Ghanaian pig populations, represented by ‘all Ghanaian pig populations’, i.e., the outliers not within the expected range; (ii) the genetic differentiation between the ADP as against all the other populations, represented by ‘ADP vs. other Ghanaian pigs’; (iii) the genetic differentiation between the exotics and other Ghanaian pig populations, represented by ‘Exotics vs. other Ghanaian pigs’; and (iv) the genetic differentiation between the crosses as against the other Ghanaian pig populations, represented by ‘Crosses vs. other Ghanaian pigs’ (Table 3). The mean *F*ST values for the regional genetic differentiations are also presented in Table 4 and Table 5. The weighted *F*_ST_ is estimated as ∑(a)/∑(a + b), where ‘a’ is the genetic variation between subpopulations and ‘b’ is the genetic variation within the population). The low mean *F*ST and weighted *F*ST values recorded across all the populations, when compared against each other either by region or by breed, suggests a low genetic differentiation between all the populations (Table 3, Table 4 and Table 5).

The *F*ST values observed within the Ghanaian population ranged between (−0.042–0.379), indicating a low-to-moderate genetic differentiation within the Ghanaian populations. Five hundred and fifty-one (551) SNPs were in the top 1% and the most significant SNPs were found within the *ANKFN1*, *ARID1A*, *ATP8A2*, *CNTN4*, *COL24A1*, *EXOC1L*, *FIGN*, *FLT1*, *FSTL5*, *IGFBP7*, *KCNH7*, *SYT9*, *UBA6*, and *UNC13B* gene regions (a complete SNP list of genes extracted can be found here: https://figshare.com/s/500c5225242c014e769c, accessed on 6 April 2021).

**Figure 4:**
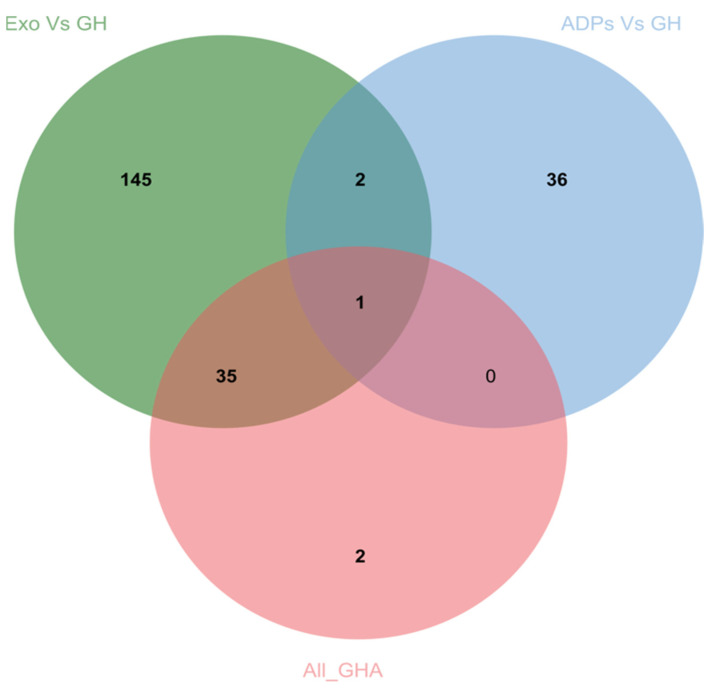
A Venn diagram showing the number of genes in common between the various Ghanaian pig populations (i.e., Exo vs. GH represents significant genes extracted from pairwise FST between the exotic population and the entire Ghanaian population, ADP vs. GH represents significant genes extracted from pairwise *F*ST between the ADP population and the entire Ghanaian population, and All_GHA represents significant genes extracted from *F*ST within the entire Ghanaian population).

When the combined ADP populations were compared against the other Ghanaian pig populations (exotic, crosses, and unknown breeds), the *F*ST values ranged between (−0.09–0.43), indicating a low-to-moderate genetic differentiation as classified by [23]. Out of the total significant SNPs, 107 SNPs were found to have *F*ST values between 0.25 and 0.4. SNPs, in the top 1% of the *F*ST values, and were considered to be under selection. For the purpose of downstream analysis, SNPs were matched to associated loci using Biomart, to allow further analysis of gene lists. The markers which recorded the highest *F*_ST_ values were found to lie within the *FAXC*, *ECSIT*, *JAZF1*, *GANT1Z*, *U6*, *ATP8A2*, *USP12*, *SPPL2C*, *PLEKHM1*, *XXYLT1*, *ERCC6*, *BTBD3*, *SPIDR*, *SHH*, *IL7*, *ST7*, *UNC5D*, *RABGAPIL*, and *THEMIS* gene regions (Table S1). A very high genetic differentiation was recorded when the exotics were compared against the other Ghanaian pig populations, i.e., ADP and crosses. The *F*ST value ranged between (−0.019–0.718), suggesting a low-to-high genetic differentiation between the exotic populations and the ADP/cross populations. SNPs within the top 1% numbered ~550 and had *F*ST values > 0.3. The highest value SNPs were found to lie within the *PDGFRA*, *RXFP1*, *FSTL5*, *PDCL2*, *ATP8A2*, *UBA6*, *GSTZ1*, *CNTN4*, *IGDCC3*, *KCNH4*, *GSTZ1*, and *FLT1* genes. The *F*ST analysis revealed that, within the Ghanaian populations, 145 SNPs were unique to the exotic populations, 36 were unique to the ADP populations, and 2 were unique to all the Ghanaian pig populations. Two genes, *DLGAP2* and *UNC5D*, were found to be shared in the top 1% between the exotic and the ADP populations and 1 gene, *ATP8A2*, was identified in all three populations (Figure 4).

Additionally, when *F*ST analysis was conducted based on regional stratification, thus comparing pig populations in the southern part versus pig populations in northern Ghana, twenty-seven (27) genes and twenty-eight (28) genes were uniquely identified in the top 1% of SNPS for the southern sector and northern sector, respectively.

**Figure 5:**
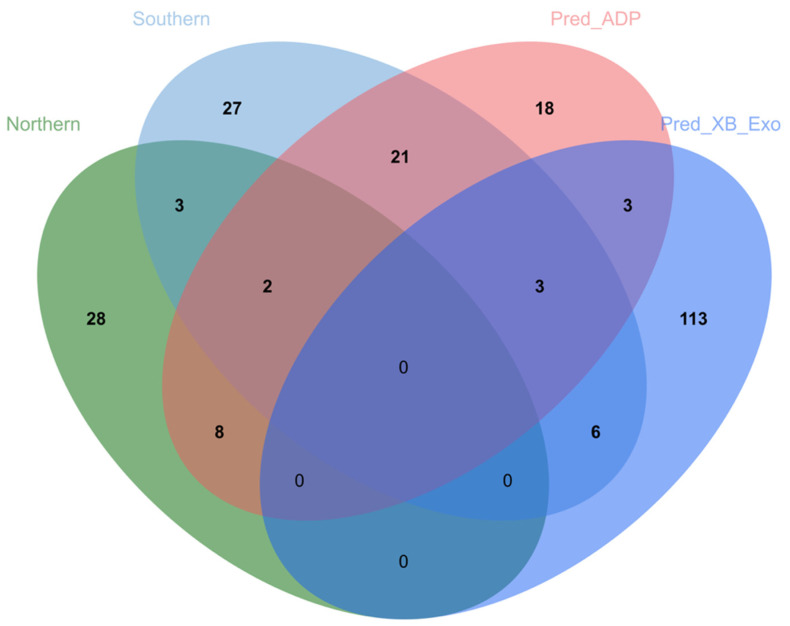
A Venn diagram showing the number of genes in common between regions with the predominant ADP (Pred_ADP) and predominant exotic/cross (Pred_XB_Exo) pig populations and northern and southern populations.

In predominantly ADP regions, 18 genes were uniquely identified, whereas in predominantly exotic and crossbred regions, 113 genes were associated with SNPs in the top 1%. Three (3) genes, *NR3C1*, *NPAS3*, and *RADIL*, were common to the southern and northern sectors. Three (3) genes, *COL14A1*, *RPL31*, and *CA10*, were found in both the predominantly ADP and predominantly cross and exotic regions. Twenty-one (21) genes are shared between the predominantly ADP regions and the southern populations and six (6) genes are shared between predominantly the exotic and southern populations. Eight (8) genes, *TRIM58*, *NEDD9*, *NUDT2*, *NDUFB6*, *ACO1*, *ANK3*, *CERS6*, and *SALL4*, are common to the northern and predominantly ADP populations, whereas no genes were found in common between predominantly exotic/cross populations and northern populations (Figure 5).

**Figure 6:**
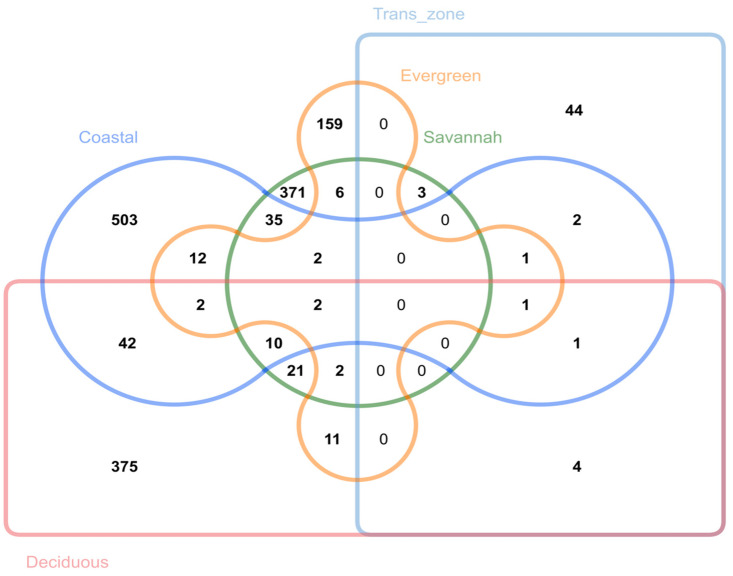
A Venn diagram showing the number of genes within the different ecological zones in Ghana and the genes shared between these ecological zones.

Within the different agro-ecological zones (see Figure 1, for agro-ecological zones’ stratification), 503 genes were found distinctively within the coastal zone, 44 genes were within the transitional zone, 159 genes were identified within the evergreen zone, 371 genes were within the savannah zone, and 375 genes were found within the deciduous zone. Among these genes, 42 common genes were identified within the coastal and deciduous zone, 21 genes within the savannah and deciduous zone, 4 genes within the transitional and deciduous zone, 12 genes within the coastal and evergreen zone, and 11 genes were found in the evergreen and deciduous zone (Figure 6).

The output of the gene ontology using String (see link for output file https://figshare.com/s/afac13db53172fa8f284, accessed on 6 April 2021) suggests that, in the savannah zone, most of the genes are related to growth, morphological, and behavioural phenotypes. It was observed that 129 of the gene counts identified are associated with body weight and measures, 166 genes are associated with protein measurements, 75 genes are associated with body height, 127 genes are associated with haematological measurements, 137 genes are associated with phenotypic abnormality, 49 genes are associated with body mass index, and 37 are associated with behavioural traits.

In the deciduous zone, again the majority of the significant genes identified are associated with mostly growth, morphological, and behavioural phenotypes. A total of 152 genes identified are associated with anthropometric measurements, 148 genes are associated with body weight and measures, 187 genes are associated with protein measurement, 81 genes are associated with body height, 85 genes are associated with bone measurement, 32 are associated with gut microbiome measurement, 56 genes are associated with body mass index, 148 genes are associated with phenotypic abnormality, 47 genes are associated with bone density, 133 are associated with haematological measurement, 21 genes are associated with facial morphology measures, and 42 genes are associated with behavioural traits. The transitional zone also has the majority of its genes involved in the regulation of biological quality (genes coloured in red) and the regulation of biological processes (genes coloured in green). Most of the genes are also involved in the positive regulation of cellular processes (genes coloured in yellow) and are highly expressed in the blood (genes coloured in blue) (Figure S1). Also, in the coastal zone, the majority of the genes identified are associated with growth and morphological traits. A total of 246 genes are associated with protein measurement, 184 genes are associated with anthropometric traits, 175 genes are associated with body weight and measures, 104 genes are associated with lipid or lipoprotein measurements, 191 genes are associated with phenotypic abnormality, 97 genes are associated with body height, and 68 genes are associated with body mass index. Finally, in the evergreen zone, the majority of the genes were associated with disease and growth phenotypes. The top significant gene cluster (35) is associated with respiratory disease biomarkers. A total of 61 genes are associated with body weight and measures, 29 genes are associated with body mass index, 77 genes are associated with protein measurements, 62 genes are associated with anthropometric measurements, 37 genes are associated with body height, and 25 genes are associated with infectious disease traits. In the various agro-ecological zones, different genes associated with pigmentation in skin or coat colour were also identified. The *TYRP1* and *TPCN2* genes were identified in the deciduous zone, *TYR* and *MITF* genes were found in the coastal zone, the *MYO7A* gene was found in the savannah zone, and *SLC24A4* was identified in the evergreen zone.

## 4. Discussion

In this study, a porcine 60 K SNP array was used to assess the genomic structure and genetic variability among pig populations in Ghana. To date, little information is known about the genomic structure of local Ghanaian pig populations. The pig populations used in this study include the following: the indigenous Ashanti Dwarf pig, crosses (between Ashanti Dwarf and European pigs), imported pigs (mostly of European descent), and other ‘unknown/nondescript’ pig populations. This work builds upon earlier research [3] to include regions of Ghana not previously reported.

Genetic diversity analysis allows us to assess the genetic variability that exists between populations for selection purposes and genetic improvements especially for adaptive and economically important traits. The high expected heterozygosity (H_E_) recorded in each of the pig populations in this study suggests high genetic variability which may be diminishing due to the relatively low observed heterozygosity (Ho) recorded, and it is comparable to other studies performed by [27], where Chinese and European pig breeds recorded relatively higher Ho than the H_E_ but vice versa in South African pigs [27]. Among the populations studied here, the ADP and crosses had a higher H_E_ and a lower H_O_ compared to the exotic populations. This may be attributed to the communal production practices under which these pig populations are raised where there is no structured record keeping for artificial insemination or mating [8,28]. This suggests the need for within-breed selection to enhance their sustainable utilisation and conservation. The results obtained in this study also suggest high inbreeding levels in the ADP and cross populations, which again may be attributed to the lack of structured mating strategies for these pig populations, where due to a lack of pedigree recording, mating is likely to occur between close relatives [6,8].

The principal component analysis demonstrates the impact of geographical locations on the clustering of pig populations in this study. The clustering separated the pig populations into two regional zones, southern and northern, with pigs in the northern part of Ghana, the Upper East, Upper West, Northern region, Ashanti, and the Brong-Ahafo region, clustering together and pigs in the southern part of Ghana, the Greater Accra, Central, Volta, Eastern, and Western regions, also clustering together. The PCA results show a strong geographical segregation of populations which could potentially be influencing the genetic compositions within the various regions as a result of the breeding strategies practised by local pig farmers in Ghana [6,8], which include the movement of pigs through trading and the act of borrowing pigs with superior traits for breeding purposes in localised communities within the different geographical regions. The results from the admixture analysis largely agree with the PCA analysis. Overall, the admixture analysis indicates that while Ashanti Dwarf pigs show some distinctiveness as a population and are clustered by region, there are notable levels of interbreeding with foreign breeds across all sampled areas. This likely stems from inadequate record-keeping regarding mating and breeding activities. Furthermore, the analysis suggests that although ADPs across various regions may share a common ancestry, variations could be attributed to diverse climatic conditions and production methods within each ecological zone [8,29].

The pairwise *F*ST genetic differentiation according to [23] recorded in the ADP population (−0.09–0.43) (Table S1) represents a low-to-high genetic differentiation at selected markers. These values were not consistent with values recorded in other village populations in South Africa and Zimbabwe [30] which recorded a low-to-moderate (0.05–0.15) genetic differentiation among their pig breeds. The low ranges recorded in the ADP populations may be attributed to the continuous gene flows between populations in nearby households who do not practise controlled breeding. However, the high genetic differentiation levels recorded at other markers may be due to the constant selection of certain alleles either unknowingly or as a result of natural selection in some of the Ashanti Dwarf populations. This could be especially true among the pigs bred at the pig research station in Babile under strict breeding and management practices. These results are similar to those observed in South African Warthog populations with moderate-to-high genetic differentiation (0.36–0.53) which were kept under restricted nature reserves that served as a physical barrier and prevented any interaction with other pig populations [27].

Across the entire sampled Ghanaian pig population, SNPs within the top 1% with a threshold of *F*ST > 0.25 were used to assess candidate genes that may be under selection and whether there are QTLs that can be associated with the top 1% of SNPs in the population. Among the peak genes identified, the genes *ANKFN1*, *ARID1A*, *ATP8A2*, and *CNTN4*, although highly expressed in the brain, have also been named to be associated with behavioural traits [31,32], growth traits [33], and reproductive traits [34], which includes abnormal eating behaviours, hyperactivity, decreased body weight, decreased embryo size, and also a positive regulation of cellular components during cellular organisation. The study conducted on local Ghanaian pigs reveals intriguing insights into the genetic factors influencing their traits, particularly in relation to their feeding behaviours and physiological responses. These pigs, predominantly raised under scavenging conditions [35], exhibit genetic markers associated with abnormal eating behaviours, decreased body weight, and reduced embryo size. Such traits could stem from their scavenging habits, wherein they ingest feeds potentially contaminated with parasites like tapeworms. Consequently, this may disrupt their antioxidant defence systems, rendering them more susceptible to stress and consequently leading to decreased performance [36]. In the genomic analysis, single nucleotide polymorphisms (SNPs) within the top 1% with a threshold of FST > 0.2 were scrutinised to identify candidate genes under selection and quantitative trait loci (QTLs) associated with these markers in the Ashanti Dwarf pig (ADP) population. Notably, a significant concentration of SNPs exceeding the threshold was found on Chromosome 4, a region known for housing QTLs linked to growth and reproductive traits [37]. Among the genes proximate to these peaks is the IL7 locus, recognised for its involvement in growth and reproductive processes in pigs [38]. Remarkably, IL7′s role in inflammatory responses [39], crucial for food digestion and nutrient absorption, has been documented in various pig populations, particularly in South African pig populations [40]. However, these responses may paradoxically dampen feed intake, consequently hindering growth rates. The presence of IL7 in the Ashanti Dwarf pig population may be attributed to their scavenging feeding behaviour. Nonetheless, it is noteworthy that inflammation responses could serve as a protective mechanism against adverse environmental conditions and heat stress, as proposed by [41]. The alleles at this locus might undergo antagonistic effects due to varying selective pressures, highlighting the complex interplay between genetic adaptations and environmental factors in shaping phenotypic traits.

The Ashanti Dwarf pig is known to be associated with a small body size, lean carcass, and low litter sizes [35,42]. Interestingly, among the significant SNPs that were identified in this study, nine genes were found to be associated with decreased body weight, decreased litter size, and a lean body. These genes include *ATP8A2*, *USP12*, *SPPL2C*, *PLEKHM1*, *XXYLT1*, *ERCC6*, *BTBD3*, *SPIDR*, and *SHH*. Genes linked with immune response were also identified in the ADP populations, such as *THEMIS*, which is known to be associated with the regulation of T-cell activation in the immune response. These genes were identified to be strongly under selection in the ADP populations and may help explain why the ADP despite being subjected to harsh production practices remains hardy.

Although the ADP populations have common ancestry, they also have a geographical stratification; it is also likely that there are genes responsible for the adaptation and differences seen between the ADPs across regions and agro-ecological zones. Particularly interestingly, different genes associated with skin or coat colour pigmentation identified in this study were uniquely correlated to the different ecological zones, which means that having a specific coat or skin colour could be much dependent on or highly influenced by the geographical location. Even though ADPs are normally considered ‘black pigs’, skin and coat colours are variable [3]. The gene *SLC24A5* identified in the evergreen zone has an allele that has been reported to be associated with a lighter skin pigmentation in human populations in East Africa [43]. This gene was also identified in African American populations where lower temperatures are predominant. The evergreen zone is characterised by lower temperatures (15–30 °C) [44]; this could mean that this gene has an allele that may have evolved due to the climatic conditions in this agro-ecological zone. The two genes *TYR* and *MITF* which were identified in the coastal zone are found in melanocytes, which are the specialised cells which produce melanin. These genes provide information to produce tyrosinase, the enzyme responsible for melanin production [45,46,47]. Allelic variants of these genes may contribute to the black pigmentation in the coat colours of the ADP populations found along the coastal belts of Ghana. In the deciduous zone, two further genes, *TPCN2* and *TYRP1*, associated with pigmentation were identified. The *TPCN2 locus* has alleles identified as being strongly associated with blond and brown hair pigmentation in humans [48]. The *TYRP1* gene, however, provides information to produce the enzyme tyrosinase-related protein 1 located in melanocytes and produces melanin. The presence of both the *TYRP1* gene and *TPCN2* gene in the list from the deciduous zones suggests that the ADPs in these zones could have alleles resulting in either brown or black coat colours (both are observed). In the savannah zone, the gene *MYO7A* has been described as responsible for providing instructions to produce the protein myosin VIIA, which is part of the group of proteins referred to as the unconventional myosins which carry melanosomes responsible for pigmentation to the cell surface. It is therefore very interesting that separate genes associated with pigmentation were identified in the various ecological zones. This explains the various colour coat patterns seen in the regional ADP populations [3] across Ghana.

## 5. Conclusions

This study has characterised the genomics of Ghanaian pig populations with a focus on the Ashanti Dwarf pig. We have also demonstrated the utility of the 60 K SNP BeadChip in assessing the genomic structure and diversity among indigenous pig populations like the ADP, despite its development for SNPs identified in European and Asian breeds. The results highlighted the genetic relatedness of all the ADP populations within the various ecological zones and regions in Ghana yet revealed considerable levels of admixture with European imported pigs as a result of indiscriminate crossing and inbreeding due to a lack of proper breed management. Putative selection signatures were also detected for regions associated with growth, coat colour, reproduction, carcass quality, and immunity traits in the Ashanti Dwarf pig population and the other pig populations in Ghana. These signatures of selections found within distinct regions and ecological zones in Ghana will be beneficial in the establishment of breeding programmes for farmers across the different regions and ecological zones within Ghana. Further genomic characterisation involving local pig genetic resources in West Africa is recommended.

## Figures and Tables

**Table 1:** Number of samples genotyped out of the samples collected from the 10 regions of Ghana.

Region	Ashanti Dwarf Pigs	Exotics	Crosses	Unknown	Total
Greater Accra region	13	1	1	0	15
Western region	12	5	9	0	26
Northern region	4	2	0	4	10
Brong-Ahafo region	17	0	5	2	24
Central Region	5	2	5	0	12
Ashanti region	4	0	4	0	8
Upper East region	15	1	7	0	23
Upper West region	13	0	3	0	16
Eastern region	7	0	7	0	14
Volta region	16	0	3	0	19
Total	106	11	44	6	167

**Table 2:** Sample size, percentage polymorphic markers, minor allele frequencies, and within-population diversity indicators calculated for each breed and entire populations.

Population	*n*	%SNP > 0.05	MAF ± SD	H_O_ ± SD	H_E_ ± SD	*F*_IS_ ± SD
ADP	106	0.88	0.26 ± 0.15	0.28 ± 0.14	0.34 ± 0.16	0.15 ± 0.17
Exotics	11	0.83	0.25 ± 0.15	0.31 ± 0.19	0.32 ± 0.17	0.05 ± 0.11
Crosses	44	0.87	0.26 ± 0.15	0.29 ± 0.15	0.34 ± 0.16	0.14 ± 0.12
Unknown	6	0.77	0.23 ± 0.16	0.30 ± 0.23	0.31 ± 0.18	0.02 ± 0.06
All	167	0.88	0.26 ± 0.15	0.29 ± 0.14	0.34 ± 0.15	0.15 ± 0.15

*n*, number of individuals; MAF, minor allele frequency; %SNP > 0.05, percentage polymorphic SNPs greater than 95%; HO, observed heterozygosity; HE, expected heterozygosity; *F*IS, inbreeding co-efficient.

**Table 3:** The mean *F*ST values and the weighted *F*ST values for the different Ghanaian pig populations studied.

Populations	Number of Animals (N)	Mean *F*ST	Weighted *F*ST
All Ghanaian populations	167	0.0156	0.0166
ADP vs. other Ghanaian pigs	105	0.0119	0.0126
Exotics vs. other Ghanaian pigs	11	0.0279	0.0349
Crosses vs. other Ghanaian pigs	44	0.00684	0.00680

**Table 4:** The mean *F*ST values and the weighted *F*ST values of the genetic differentiation between the regions studied.

Populations	Number of Animals (N)	Mean *F*ST	Weighted *F*ST
Ashanti region vs. other regions	8	0.015	0.02
Brong-Ahafo vs. other regions	24	0.017	0.02
Central region vs. other regions	12	0.014	0.016
Eastern region vs. other regions	14	0.023	0.023
Greater Accra vs. other regions	15	0.049	0.062
Northern region vs. other regions	6	0.018	0.032
Upper East vs. other regions	23	0.021	0.024
Upper West vs. other regions	16	0.033	0.042
Volta region vs. other regions	23	0.040	0.049
Western region vs. other regions	26	0.030	0.035

**Table 5:** The mean *F*ST values and the weighted *F*ST values of the genetic differentiation between the southern and northern regions and regions of predominant ADPs and predominant crosses and exotics.

Populations	Number of Animals (N)	Mean *F*ST	Weighted *F*ST
Southern sector	90	0.054	0.057
Northern sector	77	0.036	0.041
Predominantly crosses and exotics	63	0.029	0.032
Predominantly ADPs	104	0.057	0.061

## Data Availability

Data are presented in the article and in Supplementary Materials.

## Supplementary Materials

The following supporting information can be downloaded at: https://www.mdpi.com/article/10.3390/ani14050792/s1, Table S1: *F*ST analysis output showing the SNPs in the top 1% in the Ghanaian pig populations. This table shows the SNP, its position in build 11.1, and the associated gene; Figure S1: The network of significant genes in the transitional zone of Ghana. Although there are not many pathway interconnections between these genes, there are very interesting functional classifications.
