# Supplementary figures and images for: Population Structure and Genomic Characterisation of the Ashanti Dwarf Pig of Ghana
Source: Animals (Basel). 2024 Mar 4;14(5):792. doi: 10.3390/ani14050792 (PMC10931351; doi:10.3390/ani14050792)

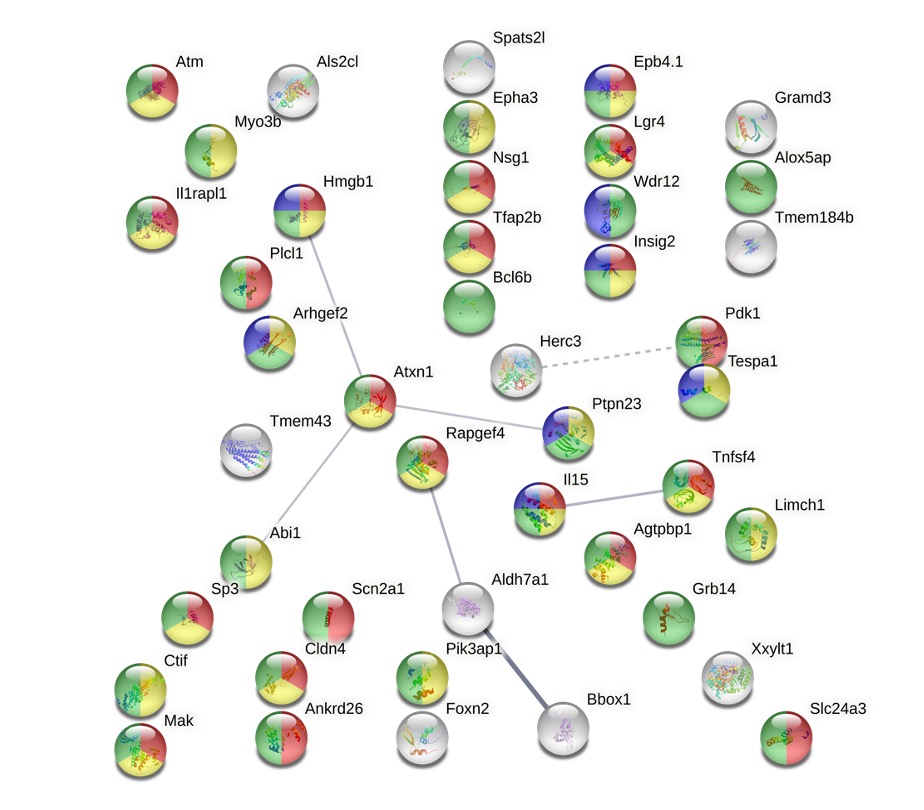

Supplement: Supplementary file 1 [file animals-14-00792-s001.zip › animals-2876094 - supplementary files/Figure S1.jpg]
